# Quercetin, a Plant Flavonol Attenuates Diabetic Complications, Renal Tissue Damage, Renal Oxidative Stress and Inflammation in Streptozotocin-Induced Diabetic Rats

**DOI:** 10.3390/metabo13010130

**Published:** 2023-01-15

**Authors:** Arshad Husain Rahmani, Mohammed A. Alsahli, Amjad Ali Khan, Saleh A. Almatroodi

**Affiliations:** 1 Department of Medical Laboratories, College of Applied Medical Sciences, Qassim University, Buraydah 52571, Saudi Arabia; 2 Department of Basic Health Science, College of Applied Medical Sciences, Qassim University, Buraydah 52571, Saudi Arabia

**Keywords:** quercetin, anti-diabetic activity, oxidative stress, reno-protective effect, anti-inflammatory activity

## Abstract

Diabetes mellitus is a metabolic syndrome characterized by increased glucose levels, oxidative stress, hyperlipidemia, and frequently decreased insulin levels. The current research was carried out for eight consecutive weeks to evaluate the possible reno-protective effects of quercetin (50 mg/kg b.w.) on streptozotocin (STZ) (55 mg/kg b.w.) induced diabetes rat models. Various physiological, biochemical, and histopathological parameters were determined in control, diabetic control, and quercetin-treated diabetic rats. The current findings demonstrated that diabetes control rats showed significantly decreased body weights (198 ± 10 vs. 214 ± 13 g) and insulin levels (0.28 ± 0.04 vs. 1.15 ± 0.05 ng/mL) in comparison to normal control. Besides this, the other parameters showed increased values, such as fasting blood glucose, triglyceride (TG), and total cholesterol levels (99 ± 5 vs. 230 ± 7 mg/dL, 122.9 ± 8.7 vs. 230.7 ± 7.2 mg/dL, 97.34 ± 5.7 vs. 146.3 ± 8 mg/dL) (*p* < 0.05). In addition, the urea and creatinine levels (39.9 ± 1.8 mg/dL and 102.7 ± 7.8 μmol/L) were also high in diabetes control rats. After 8 weeks of quercetin treatment in STZ-treated animals, body weight, insulin, and fasting blood sugar levels were significantly restored (*p* < 0.05). The inflammatory markers (TNF-α, IL-6, and IL-1β) were significantly increased (52.64 ± 2, 95.64 ± 3, 23.3 ± 1.2 pg/mL) and antioxidant enzymes levels (SOD, GST, CAT, and GSH) were significantly decreased (40.3 ± 3 U/mg, 81.9 ± 10 mU/mg, 14.2 ± 2 U/mg, 19.9 ± 2 μmol/g) in diabetic rats. All the parameters in diabetic animals treated with quercetin were restored towards their normal values. Histopathological findings revealed that the quercetin-treated group showed kidney architecture maintenance, reduction of fibrosis, and decreased expression of COX-2 protein. These results determined that quercetin has reno-protective effects, and conclude that quercetin possesses a strong antidiabetic potential and might act as a therapeutic agent in the prevention or delay of diabetes-associated kidney dysfunction.

## 1. Introduction

Diabetes is one of the main public health concerns worldwide. Type 2 diabetes mellitus accounts for more than 90% of all diabetes cases and is a chronic metabolic disease of multi-factorial origin [1]. Hyperglycemia is a chief contributor to the overall oxidative stress that leads to the production of reactive oxygen species (ROS) [2,3]. Furthermore, increased levels of ROS resulting from hyperglycemia disturbs the insulin signaling cascades and encourages the development of insulin resistance [4,5]. In addition, lipid abnormalities are prevalent in diabetes mellitus due to insulin resistance or metabolic disturbances that affect key enzymes and pathways of lipid metabolism [6]. Diabetic dyslipidemia is generally measured by higher serum levels of cholesterol and lower levels of HDL-cholesterol and triglyceride [7,8].

Several approaches are used for diabetes treatment: via the intake of healthy food and diet control, using insulin injections, or standard hypoglycemic chemical drugs. These factors increase pancreatic islet survival in addition to the regeneration of β-cells through islet neogenesis-related proteins [9,10]. The current modes of treatment for this disease may be effective but lead to adverse complications. It is common to anticipate the effectiveness of traditional herbal medicines in the prevention and treatment of diabetes with minimum or no side effects [11]. Consequently, for the treatment of diabetes mellitus a significant number of medicinal plants have been preferred as a natural source of drugs [12] as they are considered to be safe, less toxic, and more readily available than synthetic drugs [13].

In this regard, quercetin is a vital polyphenolic flavonoid present in vegetables and fruits and its role in promoting health has been demonstrated previously [14,15]. Quercetin consumption has been confirmed to affect energy production, mitochondrial biogenesis, electron transport chain performance, modification of reactive oxygen production, and mitochondrial defects [16,17]. Furthermore, its role as an anti-inflammatory, antioxidant, and anti-angiogenesis was proven in the previous study [18].

It was described that pre-treatment with quercetin protected hippocampal CA1 pyramidal neurons from ischemic injury [19]. Cigarette smoking damages human osteoblasts via the accumulation of ROS. Quercetin can reduce this damage by scavenging the radicals and upregulating the expression of HO-1 and SOD-1 [20]. It was reported that quercetin had a role in the restoration of antioxidant enzyme activity in kidney tissue of Diclofenac-treated rats. Furthermore, in the presence of quercetin, Diclofenac was unable to enhance the expression of pro-inflammatory cytokines, advocating that quercetin may have anti-inflammatory potential [21]. Its role in cancer has been documented through the modulation of various biological activities [22].

In the present study, the protective role of quercetin on streptozotocin (STZ)-induced renal damage in rats was examined via oxidative stress, lipid profile, and inflammation. In addition, histopathological analysis was performed to evaluate kidney tissue damage amongst treatment group animals.

## 2. Materials and Methods

### 2.1. Chemicals

Streptozotocin (STZ) (S0130), and quercetin (Q4951) were purchased from Sigma-Aldrich Inc., St. Louis, MO, USA. The kits of catalase (ab83464), superoxide dismutase (SOD) (ab65354), and glutathione S-transferases (GST) (ab65326) were purchased from Abcam, U.K. Inflammatory markers (TNF-α (ab46070), IL-1β (ab100767), and IL-6) (ab119548) ELISA based kits were also procured from Abcam, UK. Myeloperoxidase (MPO) (ab105136) and Nitric oxide assay kit (ab65328) were also procured from Abcam, UK. Trichrome Stain Kit (Connective Tissue Stain) (ab150686) and Picro Sirius red stain kit (ab150681) procured from Abcam, UK. H&E Staining Kit (Hematoxylin and Eosin) kit (ab245880), COX-2 primary antibody (ab15191), Mouse and Rabbit Specific HRP/DAB (ABC) Detection IHC kit (ab64264) was purchased from Abcam, UK. All supportive chemicals used in this study were of high purity grade.

### 2.2. Animal Ethics

The animal treatment procedures followed the guidelines provided by the animal care unit of CAMS, Qassim University (QU). The study was approved by the Laboratory Animal Ethics Committee (ethics committee no. 2019-2-2-I-5623) of the QU. All protocols were followed to minimize the rats’ suffering.

### 2.3. Animals and Treatment

Adult male Wistar albino rats (180–220 g) were obtained from King Saud University, Saudi Arabia. The animals were housed in plastic cages at the central animal facility unit of the College of Applied Medical Science (CAMS), Qassim University. All rats had free access to rat chow and tap water throughout the study. All animals were handled/treated in accordance with the guidelines of the Committee for the Control and Supervision of Experiments on Animals, CAMS, Saudi Arabia. The animals were grouped as 8 animals/group and were treated as described below.

The animals were rested for 7 days to reduce any transportation stress. Streptozotocin was freshly prepared in 0.05 M sodium citrate buffer (pH 4.5). The diabetes was induced by injecting intraperitoneally STZ (55 mg/kg b.w.) [23] solution in all animals except the normal control. Quercetin (50 mg/kg b.w.) was prepared in 1% dimethyl sulfoxide (DMSO) solution and was given orally by gavage to the treatment animals. Vehicle and quercetin treatments were given twice weekly after one week of diabetes induction [24], and were continued for eight consecutive weeks. The positive control rats were also given glibenclamide (5mg/kg b.w.) twice weekly. The STZ-induced rats were considered to have hyperglycemia when their fasting blood glucose levels were more than 200 mg/dL.

### 2.4. Animal Groups and Treatment Plan


**Group Name**

**Short Name**

**Treatment Plan**
Normal controlCRats with free access to rat pellets and orally given saline as a placeboNegative controlNCSTZ-induced diabetic rats at 55 mg/kg b.w. [23] and orally given saline.Positive controlPCSTZ-induced diabetic rats and oral gavage treatment with glibenclamide (5 mg/kg b.w.) [25] as a standard drug.Quercetin Treatment QTSTZ-induced diabetic rats and oral gavage treatment with quercetin (50 mg/kg b.w.)

### 2.5. Measurement of Body Weight

In all four groups, the body weights of the rats were measured weekly to check for changes in their overall weight, and the results were analyzed accordingly.

### 2.6. Fasting Blood Glucose and Insulin Level Measurement

The fasting blood glucose (FBG) was checked weekly in all experimental rats after overnight fasting, throughout the treatment plan. Blood samples were obtained from the tail vein, and FBG was measured through a standard glucometer. The level of insulin was checked at the end of the experimental design.

### 2.7. Measurement of Lipid Profile 

After overnight fasting was completed, blood samples were obtained from each rat. The serum was isolated using centrifugation at 400× *g* for 10–12 min. The total cholesterol (TC), high-density lipoprotein cholesterol (HDL-C), and triglycerides (TG) were measured accordingly.

### 2.8. Oral Glucose Tolerance Test (OGTT) 

After continuous treatment for 8 weeks, the OGTT was performed. For OGTT, fasting rats were orally given glucose at a dose of 2 g/kg b.w. Blood samples were obtained from the tail vein at different intervals such as 0, 30, 60, 90, and 120 min after administration. The blood glucose level was measured and the results were analyzed.

### 2.9. Measurement of Kidney Function Parameters

The kidney function parameters were determined by the estimation of urea and creatinine levels in serum samples from all the experimental animals and the results were interpreted accordingly.

### 2.10. Measurement of TNF-α, IL-6 and IL-1β Pro-Inflammatory Parameters

Enzyme-Linked Immunosorbent Assay (ELISA) is an in vitro enzyme-linked immunosorbent test for the quantitative evaluation of different inflammatory markers. The experiment was accomplished as per the manufacturer’s instructions for the evaluation of TNF-α, IL-6, and IL-1β levels, and the absorbance was measured at 450 nm. 

### 2.11. Measurement of Lipid Peroxidation

Malondialdehyde (MDA) was assessed via thiobarbituric acid (TBA) reactive substance at high temperature, forming a colored complex according to manufacturer guidelines. The absorbance of the resultant product was measured at 532 nm.

### 2.12. Determination of SOD, GST, CAT and GSH Levels

Kidney tissue was taken from all groups of rats and kept in a phosphate buffer saline solution. Furthermore, the tissue samples were homogenized as well as centrifuged at 1100× *g* for 15 min. Blood samples were obtained and the serum was isolated by centrifugation at 400× *g* for 12 min. The antioxidant enzymes were measured as per the kit guidelines.

### 2.13. Histopathological Examination

For microscopic evaluation, kidney tissue samples were fixed in a 10% formalin. Tissues were processed using an automated tissue processor. Paraffin was used to embed the tissue, and paraffin-embedded blocks were made for sectioning. The tissue samples were sectioned at 5 μm using a rotatory microtome. Haematoxylin/eosin (H&E) staining was performed to stain the sections. Two independent pathologists evaluated the slides in a blinded manner. H&E staining images were analyzed under a light microscope (Nikon Corporation, Tokyo, Japan) and the results were interpreted accordingly. 

### 2.14. Masson’s Trichrome Staining

The collagen fiber deposition was evaluated using Masson’s trichrome staining kit. Briefly, kidney sections were deparaffinized using xylene and properly hydrated in distilled water. Bouin’s fluid was preheated in a water bath to almost 60 °C in a fume hood and slides were placed in preheated Bouin’s fluid for one hour followed by a 10 min cooling period. Sections were cleared in distilled water. Equal parts of Weigert’s (A) and Weigert’s (B) solutions were mixed properly and slides were stained with working Weigert’s Iron Haematoxylin for 5–6 min. Slides were rinsed in running tap water for 2 min. Biebrich Scarlet/Acid Fuchsin solution was applied to each slide for 15 min. All slides were properly rinsed in distilled water. Phosphomolybdic/Phosphotungstic acid solution was used to differentiate. Aniline blue solution was applied to slides for 5–10 min and rinsed in distilled water. Acetic Acid Solution (1%) was applied to slides for 3–5 min and dehydrated rapidly in 95% alcohol, followed by changes of absolute alcohol. All slides were cleared using xylene and mounted using mounting media. The resultant formation of blue stains from the collagen deposition was examined. Two independent pathologists evaluated the slides in a blinded manner. Masson’s trichrome staining images were analyzed under a light microscope and the results were interpreted accordingly. 

### 2.15. Picro Sirius Red Staining

The fiber deposition was evaluated using the Picro sirius red staining kit of Abcam, UK. Sections were deparaffinized and hydrated with distilled water. Adequate Picro sirius red solution was applied to fully cover the tissue sections and incubated for one hour. Slides were rinsed for 2 changes of acetic acid solution. Slides were rinsed in alcohol and dehydrated in absolute alcohol. Slides were cleared, mounted, and examined accordingly.

### 2.16. Expressional Evaluation of COX-2 Protein through Immunohistochemical Staining

Briefly, formalin-fixed paraffin-embedded tissue sections were deparaffinized using xylene, rehydrated, and washed in phosphate-buffered saline (pH 7.0), and the remaining protocols were followed as per the method described earlier [26,27]. The COX-2 protein of Abcam, Cambridge, U.K. was used as primary antibodies and incubated for 1 h at 4 °C, followed by incubation with the secondary antibody for 60 min, then streptavidin–biotin enzyme complex for 1 h. Diaminobenzidine (DAB) (Abcam, Cambridge, UK, ab 64259) chromogen was then applied accordingly, and hematoxylin was used as a counterstain. Finally, the results were analyzed under a light microscope.

### 2.17. Statistical Analysis

The values are described as the means ± standard deviation. For assessments in multiple groups, one-way analysis of variance (ANOVA) was performed accordingly. The *p* < 0.05 was considered statistically significant.

## 3. Results

### 3.1. Quercetin Effects on Body Weight 

The effect of orally given quercetin on the body weight of rats was examined. Diabetic control rats showed a reduction in body weight as compared to non-diabetic rats (Table 1). At the end of 8 weeks of continuous treatment, the body weight of the rats in the normal control, diabetic control plus quercetin, and diabetic control plus glibenclamide increased significantly.

### 3.2. Effect of Quercetin on Glucose and Insulin Levels

**Figure 1:**
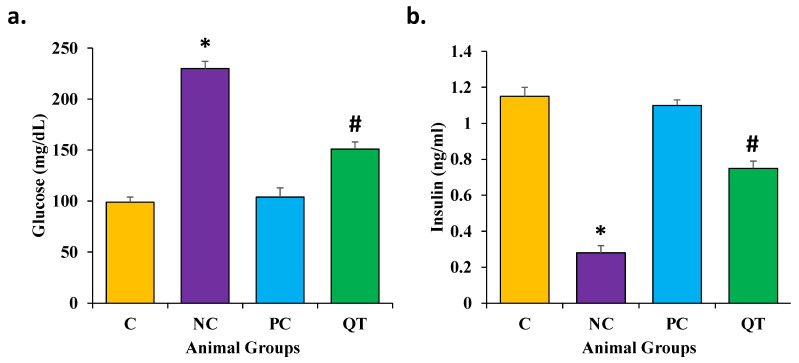
The concentration of (**a**) glucose and (**b**) insulin in serum in various animal groups after 8 weeks of continuous treatment. The animals were proportionally divided into each group (n = 8 animals per group). The data is described as mean ± SEM. * *p* < 0.05 (significant difference of b.w. (final) between NC vs. C), ^#^ *p* < 0.05 (significant difference of b.w. (final) between negative control (NC) vs. Quercetin Treatment (QT)).

The glucose and insulin levels were measured in all experimental groups. The diabetic control rats revealed high FBG levels (230 ± 7.2 mg/dL) and low insulin levels (0.28 ± 0.04 ng/mL) as compared with normal control rats (Figure 1, Supplementary File S1). Moreover, the diabetic control animals treated with quercetin showed decreased FBG levels (151 ± 6.8 mg/dL) and increased insulin levels (0.75 ± 0.06 ng/mL). However, based on these findings, it was revealed that quercetin plays a vital role in the inhibition of kidney pathogenesis through the regulation of glucose and insulin levels.

### 3.3. Effect of Quercetin on Oral Glucose Tolerance Tests

**Figure 2:**
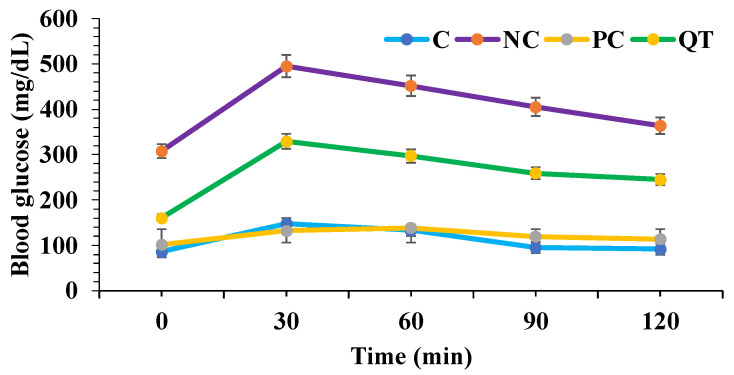
Effect of quercetin (50 mg/kg b.w.) on oral glucose tolerance test. Data are described as mean ± SEM (*n* = 8).

The effect of quercetin on diabetic rats was measured through oral glucose tolerance tests. Glucose solution (2 g/kg b.w.) was administrated by oral gavage feeding. After oral glucose intake, compared with normal control rats, diabetic rats exhibited higher blood glucose levels. Quercetin supplementation was established to improve glucose tolerance (Figure 2). 

### 3.4. Effect of Quercetin on Lipid Profile

**Figure 3:**
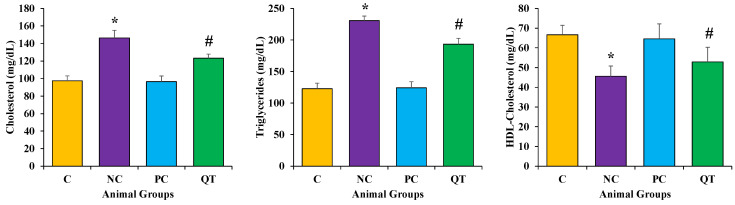
The measurement of lipid profile in different animal groups. The animals were proportionally divided into each group (n = 8 rats/group). The data is described as mean ± SEM. * *p* < 0.05 (significant difference of b.w. (final) between NC vs. C), ^#^ *p* < 0.05 (significant difference of b.w. (final) between negative control (NC) vs. quercetin Treatment (QT)).

The serum levels of triglycerides (TG), total cholesterol (TC), and high-density lipoprotein cholesterol (HDL-C) were measured in experimental animals in each group. STZ treatment showed a significant increase of TC (146.37 ± 8.7 mg/dL), TG (230.7 ± 7.2 mg/dL), and a reduction in HDL-C levels (45.6 ± 7.2) as compared to the normal control rats (*p* < 0.05 (Figure 3, Supplementary File S1). Moreover, a significant reduction in TC (123.23 ± 4.7 mg/dL), TG (193.23 ± 9.4 mg/dL), and elevation of HDL-C (52.9 ± 9.4 mg/dL) was observed in diabetic rats treated with quercetin (*p* < 0.05).

### 3.5. Effect of Quercetin on Kidney Function Profile

**Figure 4:**
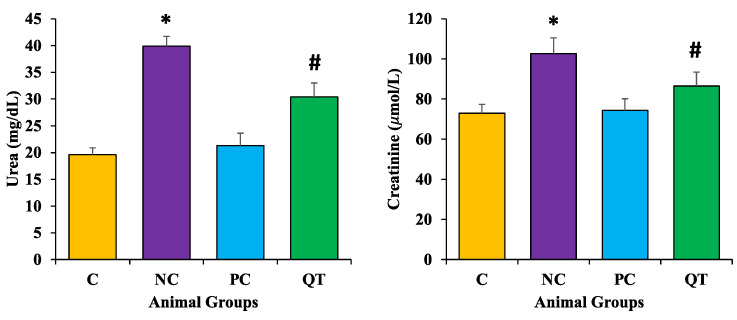
The level of urea and creatinine in different animal groups. The animals were proportionally divided into each group (n = 8 rats/group). The data is described as mean ± SEM. * *p* < 0.05 (significant difference of b.w. (final) between NC vs. C), ^#^ *p* < 0.05 (significant difference of b.w. (final) between negative control (NC) vs. quercetin Treatment (QT)).

The serum levels of urea and creatinine were measured in experimental animals in each group. STZ treatment showed significant increases in these parameters (urea as 39.9 ± 1.8 mg/dL; creatinine as 102.7 ± 7.8 μmol/L) as compared to the normal control rats (*p* < 0.05) (Figure 4). However, a significant reduction in urea (18.6 ± 2.6 mg/dL) and creatinine levels (81.5 ± 6.9 μmol/L) were observed among the diabetic animals treated with quercetin (*p* < 0.05).

### 3.6. Effect of Quercetin on Oxidative Stress

**Figure 5:**
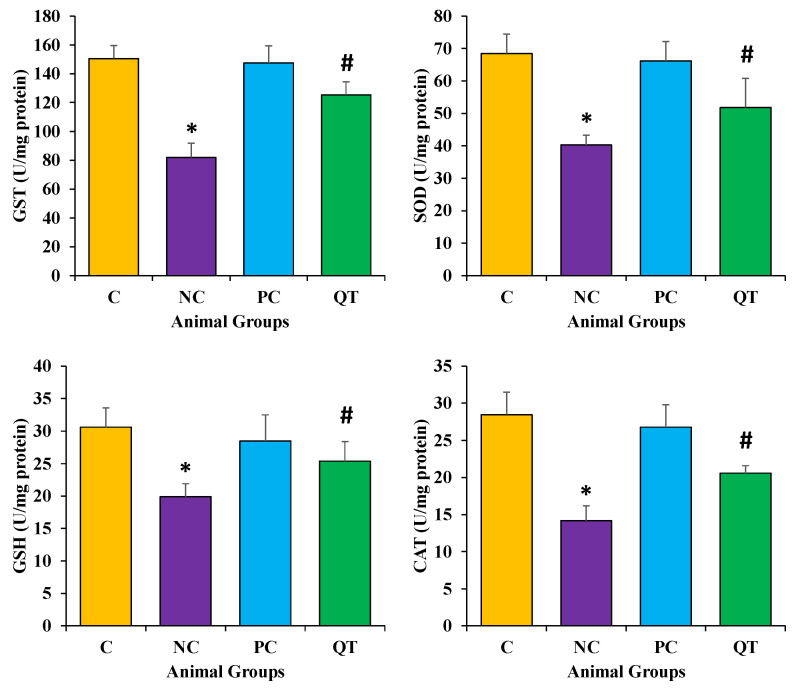
The antioxidant enzymes and antioxidant profile among different animal groups. The animals were equally divided (n = 8 animals/group). * *p* < 0.05 (significant difference of b.w. (final) between NC vs. C), ^#^ *p* < 0.05 (significant difference of b.w. (final) between negative control (NC) vs. Quercetin Treatment (QT)).

Antioxidants enzyme (CAT, SOD, GST) and GSH levels were measured to evaluate the antioxidant potential of quercetin. The results revealed that diabetic rats showed a decrease in these enzyme levels (CAT as 14.2 ± 2.2 U/mg protein, SOD as 40.3 ± 3.2 U/mg protein, GST as 81.9 ± 10.1 mU/mg protein). However, in diabetic rats treated with quercetin at doses of 50 mg/kg b.w., it showed a prominent protective effect in kidney function. The results revealed significantly increased levels of these parameters (CAT as 20.6 ± 1.1 U/mg protein, SOD as 51.8 ± 9.2 U/mg protein, and GST as 125.4 ± 8.2 mU/mg protein) after the quercetin treatment for eight consecutive weeks (Figure 5, Supplementary File S1).

### 3.7. Effect of Quercetin Extract on Lipid Peroxidation

**Figure 6:**
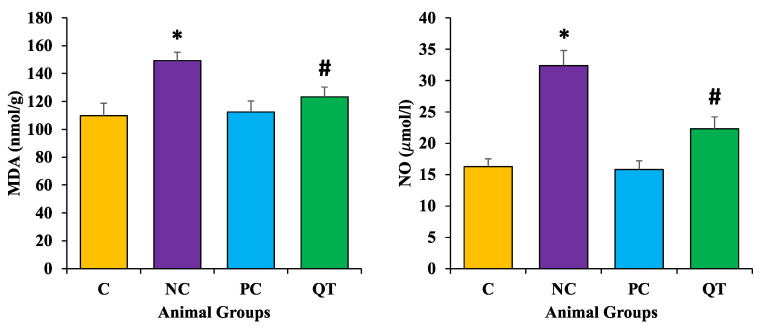
The malondialdehyde and nitric oxide levels in different animal groups. The animals were equally divided (n = 8 animals/group). * The data is described as mean ± SEM. * *p* < 0.05 (significant difference of b.w. (final) between NC vs. C), ^#^ *p* < 0.05 (significant difference of b.w. (final) between negative control (NC) vs. Quercetin Treatment (QT)).

Malondialdehyde (MDA) and nitric oxide (NO) content were measured to examine the protective role of quercetin on oxidative stress. Results revealed that diabetic rats showed enhanced levels of both MDA (149.3 ± 3.2 nmol/g) and NO (32.4 ± 2.4 μmol/L) content as compared to the control rats. However, the diabetic rats treated with quercetin showed a prominent reduction of these parameters (MDA as 123.3 ± 7.2 nmol/g and NO as 22.3 ± 1.9 μmol/L) (Figure 6). 

### 3.8. Effect of Quercetin on Inflammatory Markers Level

**Figure 7:**
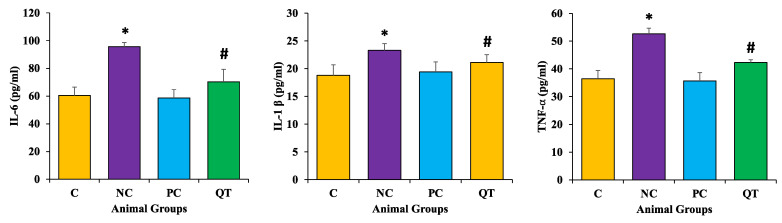
The level of different inflammatory markers in various animal groups. The animals were proportionally divided into each group (n = 8 rats/group). The data is described as mean ± SEM. * *p* < 0.05 (significant difference of b.w. (final) between NC vs. C), ^#^ *p* < 0.05 (significant difference of b.w. (final) between negative control (NC) vs. quercetin Treatment (QT)).

The levels of InterlukinL-6, TNF-α, and IL-1β levels were raised significantly in diabetic control rats (IL-6 as 95.64 ± 3.2 pg/mL, IL-1β as 23.30 ± 1.2 pg/mL, and TNF-α as 52.64 ± 2.2 pg/mL) when compared to the control group (*p* < 0.05). The treatment of diabetic rats with quercetin significantly decreased the level of these pro-inflammatory markers (IL-6 as, 70.29 ± 9.3 pg/mL, IL-1β as 21.10 ± 1.4 pg/mL, and TNF-α as 42.29 ± 1.3 pg/mL) towards the normal levels (*p* < 0.05) (Figure 7).

### 3.9. Effect of Quercetin on Kidney Histology

**Figure 8:**
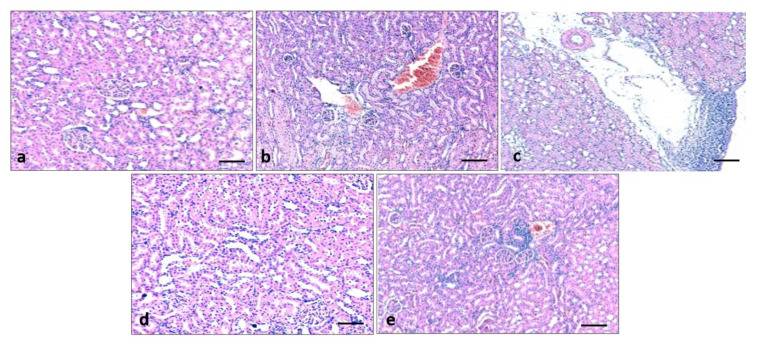
Effect of quercetin extract in kidney tissue architecture. Light photomicrographs of kidney sections from the control group: normal architecture was noticed (**a**), streptozotocin (STZ) group (Negative control): showed infiltration of lymphocytes, and congestion (**b**,**c**), STZ + glibenclamide (Positive control) showed normal kidney architecture (**d**), STZ + quercetin (50 mg/kg b.w.) displayed mild inflammation and congestion (**e**).

To evaluate the effect of quercetin on renal structural changes in all experimental animals, renal morphology was examined through Haematoxylin and Eosin staining. Histological study of normal control groups showed normal renal architecture as normal glomerulus, proximal and distal tubules with normal epithelium. The histopathological sections of kidneys in diabetic rats showed distorted glomerular morphology, congestion, and infiltration of lymphocytes, demonstrating kidney injury. On the other hand, pathological changes of the kidney tissues in diabetic groups that received quercetin showed less damage as less congestion and fewer inflammatory cells were observed as compared to the diabetes control group (Figure 8).

### 3.10. Effect of Quercetin on Renal Fibrosis

**Figure 9:**
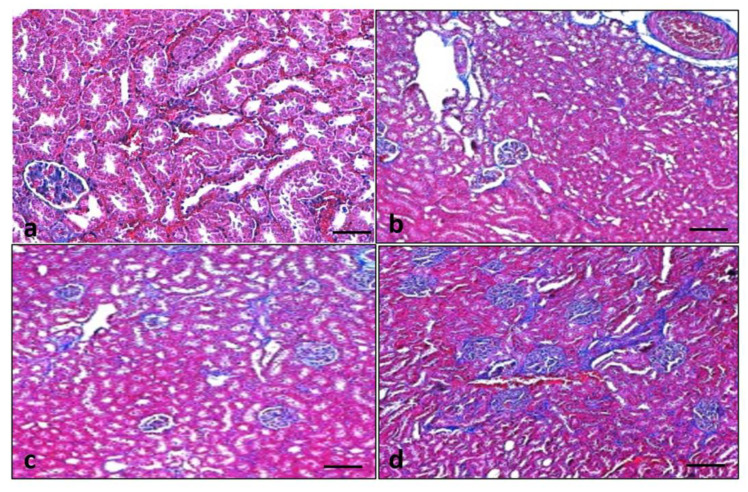
Quercetin decreases collagen fiber in the kidney tissues. Light photomicrographs of kidney sections from the control group: collagen fiber not seen (**a**), streptozotocin (STZ) group (Negative control): high deposition of collagen fiber noticed (**b**), STZ + glibenclamide (Positive control) collagen fiber was significantly less (**c**), STZ + quercetin (50 mg/kg b.w.): showed less collagen fiber (**d**).

Collagen fiber was examined using Masson’s trichrome staining to detect the effects of quercetin on renal fibrosis and changes in collagen fiber in the control and experimental groups. Furthermore, this staining established that collagen fiber (blue staining) was significantly high in diabetic rats. Quercetin treatment (50 mg/kg) reduced the collagen deposition in the renal tissue of diabetic rats when compared to the STZ-induced diabetes group (Figure 9).

### 3.11. Effect of Quercetin on Renal Fibrosis of STZ-Induced Diabetic Rats

**Figure 10:**
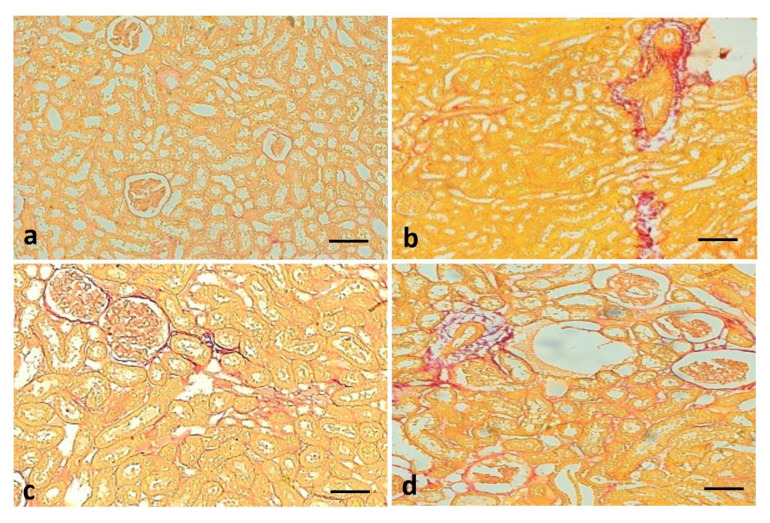
Quercetin decreases fiber in renal tissue. Kidney sections from the control group: did not show fibrosis (**a**), streptozotocin (STZ) group (Negative control): deposition of fiber (red color) was significantly high (**b**), STZ + glibenclamide (Positive control) showed almost no fibrosis (**c**), STZ + quercetin (50 mg/kg b.w.): showed less fiber as compared to streptozotocin (STZ) group (Negative control) (**d**).

Picro sirius staining was performed on all experimental groups to evaluate the reno-protective effect of quercetin. Picro sirius staining demonstrated that fibrosis (red staining) was significantly high in the disease control group (diabetic rats). Quercetin treatment (50 mg/kg) plays a reno-protective role through the reduction of fibrosis in the renal tissue of diabetic rats when compared to the STZ-induced diabetes group (Figure 10).

### 3.12. Effect of Quercetin on COX-2 Protein Expression of STZ-Induced Diabetic Rats

**Figure 11:**
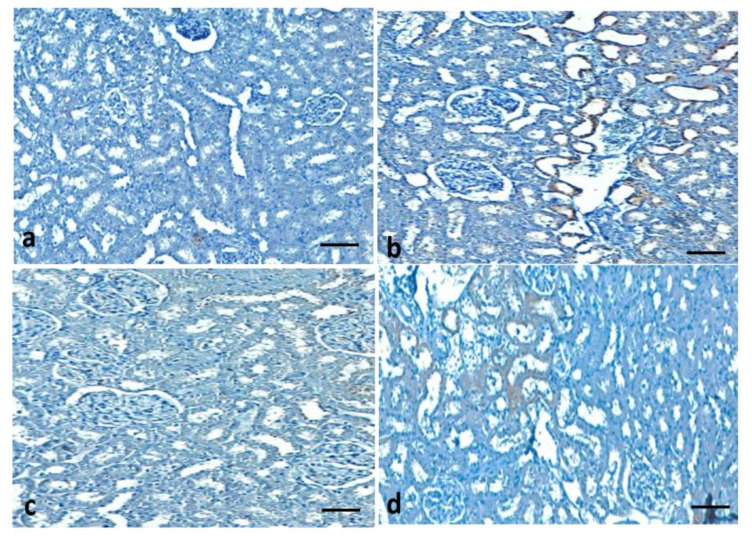
Quercetin decreases COX-2 protein in the renal tissues. Kidney sections from the control group: did not show any expression (**a**), streptozotocin (STZ) group (Negative control): expression of COX-2 protein was high (**b**), STZ + glibenclamide (Positive control) fiber showed almost no expression (**c**), STZ + quercetin (50 mg/kg b.w.): showed less expression as compared to the streptozotocin (STZ) group (Negative control) (**d**).

Immunohistochemistry staining was performed on all experimental groups to evaluate the COX-2 protein expression. The expression of COX-2 was high in the diseases control group (diabetic rats). Quercetin treatment (50 mg/kg) plays a reno-protective role through the decrease of COX-2 protein expression in the renal tissue of diabetic rats when compared to the STZ-induced diabetes group (Figure 11).

## 4. Discussion

Diabetes is a metabolic disorder and is characterized by chronic hyperglycemia with altered metabolism of carbohydrates, proteins, and fats resulting from altered insulin secretion, action, or both [28]. Hyperglycemia is one of the main contributors to the production of ROS and oxidative stress [2,3]. In addition, increased levels of ROS also disturb the cascade of insulin-signaling, inspiring the development of insulin resistance [3,4]. Therefore, the recognized altered state is associated with ROS over-production which causes a state of oxidative stress that is involved in the pathogenesis as well the progression of diabetes and diabetes-linked complications [29].

Natural compounds, as whole or specific active compounds of medicinal plants, play an important role in the inhibition of pathogenesis including diabetes [30,31,32,33]. To know whether quercetin has a role in the management of diabetes mellitus, it was examined by checking its role as an anti-hyperglycemic, oxidative stress, anti-inflammatory activity, and in kidney tissue architecture at a dose of 50 mg/kg b.w. in rats. STZ-induced diabetic rats treated with quercetin (50 mg/kg b.w.) showed a significant increase in body weight. All animals in the diabetic control group showed a decrease in body weight when compared to the normal control and STZ-induced diabetic rats treated with quercetin. Our results are consistent with those of earlier studies and it was reported that STZ-induced diabetes is characterized by a major loss in body weight resulting from increased muscle destruction or degradation of structural proteins [34]. Moreover, the treatment with quercetin in STZ-induced diabetic rats prevented the changes in body weight and blood glucose [35]. In addition, quercetin improved the decrease in body weight gain, and it possibly prevented reductions in body weight gain and polyuria [36]. This result is justified by previous findings as better glycemic control, with a reduction of the lipolytic response, and the consequential normalization of triglyceridemia [37,38].

After the oral administration of quercetin, the STZ-induced diabetic rats showed a significant decrease in blood glucose levels and increased insulin levels. These results are in accordance with earlier findings of antidiabetic studies that reported elevated serum blood glucose levels and insulin levels in diabetic rats were significantly improved by quercetin, resveratrol, and combined treatments [39].

It is well recognized that uncontrolled type 2 diabetes mellitus leads to increased triglycerides, *LDL* and VLDL-C, and decreased HDL-C, which promotes coronary artery diseases [40,41]. Furthermore, it has been shown that insulin deficiency in diabetes mellitus leads to a variety of derangements in metabolic and regulatory processes, which in turn lead to the accumulation of lipids like triglycerides and TC in diabetic patients [42]. In the current study, STZ treatment showed a significant increase in total cholesterol, triglycerides, and a reduction in HDL-C levels as compared to the normal control rats. Moreover, a significant reduction in TC, TG, and an elevation of HDL-C was noticed in diabetic rats treated with quercetin. Earlier findings were in agreement with the current findings and the study revealed that administration of quercetin showed significant improvements in the profiles of high-density lipoprotein, triglycerides, and total cholesterol in STZ-induced diabetic rats [43].

Streptozotocin causes major oxidative stress in diabetic animals and possibly causes the peroxidation of polyunsaturated fatty acids, important for the formation of MDA. which is a by-product of lipid peroxidation [44]. Oxidative stress plays a major role in the disturbance of cellular functions in the kidney and causes vascular permeability enhancement and tissue damage [45]. This suggestion is evidenced by the previous results [38,46] which show indications of renal oxidative stress in diabetic rats [47,48,49,50].

Measurements of MDA as a final product of the lipid peroxidation reveal the degree of oxidative stress [51]. STZ injections were administered to illustrate the cellular oxidative damage as it produces ROS and decreases the antioxidant potential in the pancreas, which is known to deteriorate this organ [52]. Antioxidants play a significant role in the reduction of MDA levels. In the current study, it is reported that diabetic rats showed a decrease in antioxidant enzymes (CAT, SOD, and GST) and GSH levels. However, in diabetic rats, quercetin treatments at doses of 50 mg/kg b.w. produced a prominent protective effect. The results revealed significantly increased levels of MDA and NO in the kidneys of diabetic rats. On the other hand, treating diabetic rats with 50 mg/kg b.w. quercetin for 8 weeks considerably improved the oxidative status through significant decreases in levels of MDA and NO, and increases in antioxidant enzyme levels. In this context, the previous study was in accord with the current findings as after 8 weeks of continuous treatment, diabetic rats displayed renal dysfunction, as confirmed by decreased urea clearance and creatinine, and proteinuria nearby with a noticeable increase in oxidative stress, as determined by lipid peroxidation. Whereas treatment with quercetin significantly reduced oxidative stress and renal dysfunction in diabetic rats [53].

STZ treatment showed a significant increase in urea and creatinine levels as compared to the normal control. However, an important reduction in urea and creatinine levels were detected among the diabetic animals treated with quercetin. The previous findings were in accordance with the current findings as quercetin treatment decreased proteinuria and high plasma levels of uric acid, urea, and creatinine [37].

Quercetin has a proven role in the inhibition of pathogenesis, including kidney damage through the maintenance of kidney architecture. The histopathological sections of kidney in diabetic rats showed congestion, fibrosis and infiltration of lymphocytes, and deposition of collagen fiber demonstrating kidney injury. Whereas pathological changes of the kidney tissue in the diabetic groups that received quercetin showed less injury and reduced fibrosis as compared to the diabetes control group. The renoprotective effect of quercetin has been proven in other studies as diabetic rats showed changes such as brush border loss and peritubular infiltration, epithelial desquamation, swelling, and intracytoplasmic vacuolization. Moreover, sclerotic changes and basement membrane thickening were seen in the glomerulus, whereas, quercetin significantly reduced such histopathological changes [48]. Other study findings were consistent with the current study’s results and it was reported that STZ-induced rats showed inflammatory cell infiltration in the renal tubular and glomerulus. These kidney pathological alterations in this model were improved by treatment with quercetin [47].

Oxidative stress can cause inflammation through numerous mechanisms [54] and inflammation played an important role in the pathogenesis of diabetes [55,56]. STZ treatment showed significant enhancement of inflammatory marker levels and COX-2 protein expression as compared to the normal control. However, a decrease in inflammatory markers was observed among the diabetic animals treated with quercetin. In other novel research work, it was reported that quercetin treatment for diabetic rats led to significant decreases in oxidative stress, inflammation, and apoptosis levels [57]

In the current study, STZ-induced diabetic rats showed significant fibrosis in the kidney tissue, which was reduced by quercetin. The results established that quercetin could improve kidney injury through the maintenance of kidney architecture and reduction of fibrosis. These results are consistent with the previous reports as quercetin and crocin inhibit renal fibrosis as confirmed by Masson trichrome staining [58]. Another study reported similar findings based on *Myrciaria cauliflora* extract as increased collagen deposition was also observed in the STZ group when compared to the control group. Furthermore, the collagen deposition was inhibited by *Myrciaria cauliflora* extract in a dose-dependent manner [59].

## 5. Conclusions

The current findings reveal the anti-diabetic, antihyperlipidemic, anti-inflammatory, and reno-protective effects of quercetin against STZ-induced diabetes. Quercetin enhanced antioxidant enzyme levels and maintained kidney architecture. This study determines that the effects of quercetin have the potential for use in the management of diabetes mellitus, however, comprehensive, detailed biochemical and molecular studies should be performed to identify the exact mechanisms of quercetin’s renoprotective effects.

## Figures and Tables

**Table 1:** Body weight of different animal groups measured at start and end of the treatment plan. The animals were divided equally among different groups (n = 8 per group). The data is represented as mean ± standard error of the mean (SEM). * *p* < 0.05 (significant difference of final body weight between NC vs. C), ^#^ *p* < 0.05 (significant difference of final boy wight between NC vs. QT).

Animal Groups	Body Weight (0 Days) (g)	Body Weight (After 8 Weeks) (g)
Control (C)	216 ± 12	295 ± 10
Negative Control (NC)	214 ± 13	198 ± 10 *
Positive Control (PC)	213 ± 11	272 ± 12
Quercetin Treatment (QT)	219 ± 12	263 ± 14 ^#^

## Data Availability

The data used to support the findings of this study are included within the article.

## Supplementary Materials

The following supporting information can be downloaded at: https://www.mdpi.com/article/10.3390/metabo13010130/s1, Supplementary File S1: supplementary data.
