# Supplementary material for: Quercetin, a Plant Flavonol Attenuates Diabetic Complications, Renal Tissue Damage, Renal Oxidative Stress and Inflammation in Streptozotocin-Induced Diabetic Rats
Source: Metabolites. 2023 Jan 15;13(1):130. doi: 10.3390/metabo13010130 (PMC9861508; doi:10.3390/metabo13010130)
Supplement: Supplementary file 1 [file metabolites-13-00130-s001.zip › metabolites-2121728-Supplementary File S1.pdf]

Supplementary data

1. Glucose and Insulin level

Groups	Glucose (mg/dl)	Insulin (ng/ml)
C	99±5.3	1.15±0.05
NC	230±7.2	0.28±0.04
PC	104±9.4	1.1±0.03
QT	151±7.1	0.75±0.04

2. Lipid profile

Groups	Cholesterol (mg/dL)	Triglycerides (mg/dL)	HDL-Cholesterol (mg/dL)
C	97.34±5.7	122.9±8.5	66.7±8.7
NC	146.37±8.7	230.7±7.3	45.6±7.2
PC	96.45±6.4	124.3±9.1	64.6±9.2
QT	123.23±4.6	193.23±9.3	52.9±9.4

3. Kidney function profile

Groups	Creatinine (μmol/L)	Urea (mg/dL)
C	72.9±4.5	19.6±1.3
NC	102.7±7.8	39.9±1.8
PC	74.39±5.3	30±2.3

QT	81.5±6.9	18.6±2.6

4. Antioxidant enzyme level

Groups	SOD (U/mg protein)	CAT (U/mg protein)	GSH (μmol/g protein)	GST (mU/mg protein)
C	68.5±6.3	28.5±3.5	30.6±3.1	150.6±9.3
NC	40.3±3.7	14.2±2.6	19.9±2.4	81.9±10.4
PC	66.2±6.2	26.8±3.7	28.5±4.1	147.5±12.6
QT	51.8±9.5	20.6±1.4	25.4±3.6	125.4±9.7

5. Lipid peroxidation status

Groups	MDA (nmol/g)	NO (μmol/L)
C	109.7±6.2`	16.3±1.2
NC	149.3±3.2	32.4±2.4
PC	112.4±6.7	15.8±1.4
QT	123.3±4.2	22.3±1.9

6. Inflammatory markers

Groups	TNF alpha (pg/ml)	IL-6 (pg/ml)	IL-1 beta (pg/ml)
C	36.49±3.2	60.49±6.2	18.80±1.9
NC	52.64±2.1	95.64±3.7	23.30±1.2
PC	35.68±3.6	58.68±6.1	19.40±1.8
QT	42.29±1.3	70.29±9.5	21.10±1.4
